# Genetic and Environmental Contributions to Variation in the Stable Urinary NMR Metabolome over Time: A Classic Twin Study

**DOI:** 10.1021/acs.jproteome.1c00319

**Published:** 2021-07-26

**Authors:** Kate M. Bermingham, Lorraine Brennan, Ricardo Segurado, Rebecca E. Barron, Eileen R. Gibney, Miriam F. Ryan, Michael J. Gibney, Aifric M. O’Sullivan

**Affiliations:** † UCD Institute of Food and health, School of Agriculture and Food Science, University College Dublin, Belfield Dublin 4, Ireland; ‡ School of Public Health, Physiotherapy and Sports Science, University College Dublin, Belfield Dublin 4, Ireland

**Keywords:** stable NMR metabolome, intra- and interindividual variations, conservation, genetic and environmental influences

## Abstract

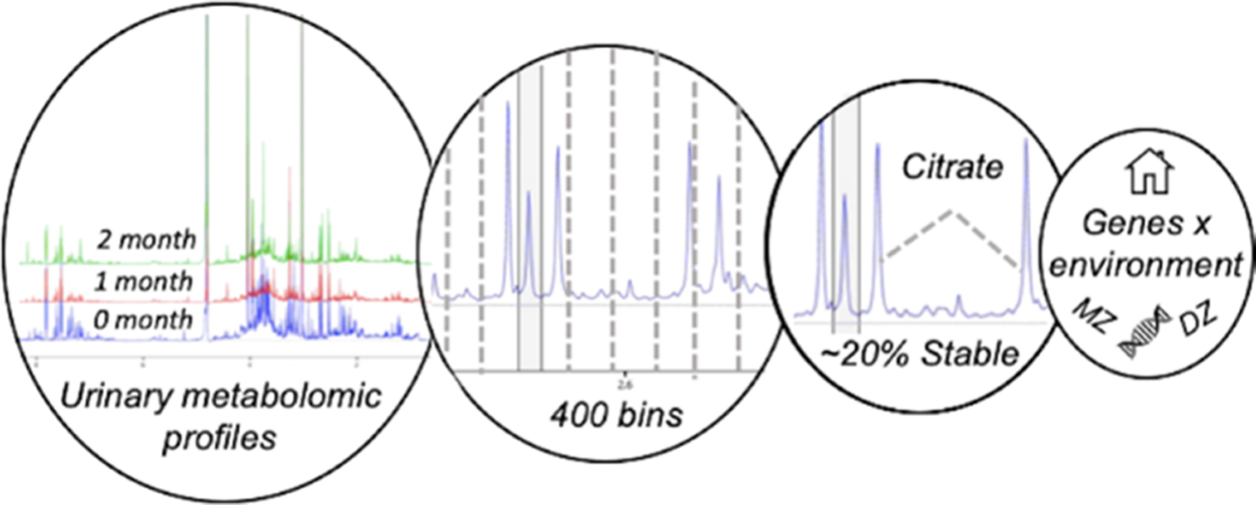

Genes, sex, age, diet, lifestyle, gut microbiome, and multiple other factors affect human metabolomic profiles. Understanding metabolomic variation is critical in human nutrition research as metabolites that are sensitive to change versus those that are more stable might be more informative for a particular study design. This study aims to identify stable metabolomic regions and determine the genetic and environmental contributions to stability. Using a classic twin design, ^1^H nuclear magnetic resonance (NMR) urinary metabolomic profiles were measured in 128 twins at baseline, 1 month, and 2 months. Multivariate mixed models identified stable urinary metabolites with intraclass correlation coefficients ≥0.51. Longitudinal twin modeling measured the contribution of genetic and environmental influences to variation in the stable urinary NMR metabolome, comprising stable metabolites. The conservation of an individual’s stable urinary NMR metabolome over time was assessed by calculating conservation indices. In this study, 20% of the urinary NMR metabolome is stable over 2 months (intraclass correlation (ICC) 0.51–0.65). Common genetic and shared environmental factors contributed to variance in the stable urinary NMR metabolome over time. Using the stable metabolome, 91% of individuals had good metabolomic conservation indices ≥0.70. To conclude, this research identifies 20% of the urinary NMR metabolome as stable, improves our knowledge of the sources of metabolomic variation over time, and demonstrates the conservation of an individual’s urinary NMR metabolome.

## Introduction

Metabolomics involves the comprehensive systematic profiling of metabolites in a biological sample.^1^ In nutrition and health research, metabolomics enhances our understanding of the effects of foods or diet on metabolic pathways and identifies dietary biomarkers.^2^ Levels and patterns of intra- and interindividual variations differ for every metabolite. To identify robust associations with a metabolite, studies must understand and control for the influences contributing to variation in metabolites over time. Metabolites that are sensitive to short-term changes in diet or lifestyle are useful in dietary intervention studies, whereas stable metabolites might provide useful information on longer-term markers of diet or health.

The metabolomic composition of biofluids is affected by many factors including gene sex, age, diet, lifestyle, and the gut microbiome.^3,4^^5,6^ Using repeated sample collections, fair to good stability over several days to 3 years has been reported for blood and urine metabolites.^3,5,7−10^ Floegel et al.^7^ reports good stability for 163 serum metabolites over a 4 month period, defining good stability as intraclass correlation (ICCs) between 0.51 and 0.74. Stable metabolites measured using flow injection analysis tandem mass spectrometry (MS) included hexose, sphingolipids, amino acids, and glycerophospholipids.^7^ In urine, 31% of 539 metabolites, measured using liquid chromatography-MS and gas chromatography-MS, had excellent stability (ICC > 0.80) over 60–90 days.^4^ Age, sex, and body mass index (BMI) explained only a small proportion of variation in the stable metabolites, suggesting that other factors contribute toward stability.^4^ Although blood is under tighter homeostatic control compared to urine, findings demonstrate that some metabolites are more stable compared to others over time across both biofluids.

Blood metabolomic profiles are characteristic of an individual and conserved over 8–10 years.^11−14^ In an Italian cohort, healthy subjects were characterized by a stable metabolic space over 10 years.^14^ Conditions including pregnancy, lactation, or cancer were associated with deviations from a stable metabolic space.^14^ Yousri et al.^15^ examined the long-term conservation of human metabolomic profiles over 7 years, showing that 53% of the cohort had excellent metabolome conservation. Highly stable metabolites increased metabolome conservation.^15^ Stable metabolites could be grouped into those that were conserved due to genetics and those conserved due to dietary or lifestyle preferences.^15^ Nicholson et al.^16^ decomposed biological variation in plasma and urinary NMR metabolites collected over 4 months into two stable (familiality and individual environment) and two unstable (individual-visit and common-visit) components. Stable components accounted for, on average, 60 and 47% of the variation in plasma and urinary metabolites, respectively.^16^ Using a classic twin design, the authors demonstrate that familial factors comprising genetic and shared environment contribute a stable and pervasive influence to stability in the NMR metabolome.^16^ Research suggests that stable metabolites may improve metabolome conservation and stable metabolites are influenced by a combination of genetic and environmental factors.^3,15^

To date, the plasma metabolomic profile has been used to examine metabolomic conservation or how well an individual is recognized with themselves over time, whereas less is known about the stability of the urine metabolome. Lower blood metabolomic profile conservation has been associated with an increase in all-cause mortality risk independent of several health parameters including cardiovascular risk factors and chronic illness.^17^ Thus, changes in metabolism could be identified by monitoring the conservation of an individual’s metabolomic profile without focusing on specific disease biomarkers. This study aims to classify stable regions of the urinary NMR metabolomic profile and quantify the genetic and environmental contributions to stability over time using a classic twin design.

## Materials and Methods

### Study Population

The UCD twin study is a semilongitudinal classic twin study. Participants included healthy male and female monozygotic (MZ) and dizygotic (DZ) twins (same sex) aged 18–65 years living in Ireland. The study design and inclusion and exclusion criteria were described previously.^18^ Briefly, participants attended five study visits over a 2 month period, with a visit approximately every 2 weeks (±3 days). Each twin pair completed their visits within 1 month of one another. Urine samples collected at baseline, 1 month, and 2 month visits were used in this research. One-hundred and twenty-eight participants constitute our study population. Ethical approval was obtained from the Human Research Ethics Committee in University College Dublin, and all participants provided informed written consent (LS-13-44-OSullivan). All procedures were conducted according to the principles expressed in the Declaration of Helsinki. A cohort of 64 twin pairs (88 MZ and 40 DZ twins) is powered to estimate additive genetic effects (A) ≥ 77% with an 80% power.^19^ The power to detect a range of significant parameters in the UCD twin study cohort was also examined (Supporting Information Table S-1). While this study is powered to estimate heritability for certain traits, the sample size is small and therefore insufficient to estimate significant contributions of genetics and environment for all traits. Figure S-1 describes the study workflow design.

### Biofluid Collection

On each study visit in the morning, after an overnight fast, participants collected a first-void midstream urine sample. Samples were placed on ice packs and transported to the study center. Samples were inverted twice and immediately centrifuged at 1500*g* for 10 min and stored at −80 °C until analysis. Buccal swabs were collected for zygosity analysis and confirmed by 21 DNA markers (Genetic Testing Laboratories Inc. Brighton, U.K.).

### NMR Spectroscopy

Spot urine samples were prepared by the addition of 250 μL of phosphate buffer (0.2 M KH_2_PO_4_, 0.8 K_2_HPO_4_ at pH 7.4), 10 μL of sodium trimethylsilyl propionate (0.05 g/4 mL), and 50 μL of D__2__0 (99.9%) to 500 μL of urine. Spectra were acquired on a 600 MHz Varian NMR spectrometer using a nuclear Overhauser spectroscopy (NOESY) pulse sequence with 16 K complex points and 128 scans over a width of 9 kHz. Water suppression was achieved during the relaxation delay (2.5 s) and mixing time (100 ms). ^1^H NMR urine spectra were processed manually with Chenomx software and were line-broadened and phase- and baseline-corrected. Spectra (10.00–0.00 ppm) were reduced by dividing the spectra into bins of 0.02 width. The area of the bin was calculated to represent the spectral region. Data were normalized to the sum of the spectral integral. Metabolomic bin regions were transformed using Johnson transformation. Metabolites within bin regions classified as “stable” were identified using Chenomx software and are presented in Supporting Information Table S-2 (Chenomx Inc., Edmonton, Canada).

### Dietary Analysis and Anthropometric Measurements

Dietary intake was assessed on 5 nonconsecutive days, over a 2 month period, using the 24 h recall method based on the U.S. Department of Agriculture Automated Multiple-Pass Method (USDA AMPM). Collection and analysis of dietary data were previously described.^20^ Briefly, food intake data were coded and entered into the WISP version 3.0 (Tinuviel Software, U.K.) for analysis. All data were quality-controlled for accuracy and assessed for under-reporters of energy intake using Henry equations.^21^ Healthy eating index (HEI)-2015 components and scores were calculated according to the criteria set out by Krebs-Smith et al.^22^ Height was measured to the nearest millimeter with a Leicester portable height measure (Chasmores Ltd., U.K.) without shoes; body mass was measured in duplicate using a Tanita body composition analyzer BC- 420MA (Tanita Ltd., U.K.), and body composition, including fat-free mass (FFM), was measured by air-displacement plethysmography (BOD-POD, Life Measurements Instruments).

### Statistical Analysis

Statistical analysis was performed using the R statistical suite version 3.6.1 for Mac OS X. Linear mixed modeling was performed to decompose total variance (σ_T_^2^) in urinary metabolomic bin regions into several components: interindividual variance (σ_B_^2^), which can also be considered the variance of the “usual” level in a population; intraindividual variance (σ_W_^2^), which reflects monthly variability around the usual level within an individual; and intrafamily variance (σ_F_^2^), which accounts for family relatedness. The three-variance components were estimated using the linear mixed modeling equation
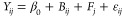
1*Y*_*ij*_ is the normalized transformed metabolomic bin level of twin *i* from pair *j* and random effects included subject ID (*B*_*ij*_), and family ID (*F*_*j*_), which represents the omitted family characteristics or unobserved heterogeneity. The intraclass correlation (ICC) is denoted as the proportion of the population’s biologic variability that is due to the interindividual variation as well as intrafamily variation, accounting for similarities between twins
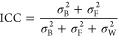
2

### Variance Explained by Age, Sex, FFM, and HEI

Equation 1 was expanded to include the four covariates, age (α_a_), sex (δ_g_), FFM (γ_w_), and HEI score (ϕ_h_)
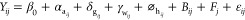
3Fixed effects for age (α_a_ij__), sex (δ_g_ij__), FFM (γ_w_ij__), and HEI score (ϕ_h_ij__) were included for each subject, i.e., twin *i* from pair *j*. We assessed whether the covariates were significantly associated with metabolomic bin regions and obtained *p*-values by conducting an analysis of variance (ANOVA) on the mixed models. The Benjamini–Hochberg correction for multiple comparisons was applied, and statistically significant thresholds were based on false discovery rate (FDR) cutoffs (*p* < 0.05).^23^ The proportion of variance explained by each significant covariate (*R*^2^) was estimated for each metabolomic bin region using the R package MuMIn.^24^ Total variance was defined as , and the proportion of the variance attributable to a significant covariate was defined as.

### Classification of Stable Metabolomic Regions

For each metabolomic bin region, significant covariates were included in eq 3. ICC values were calculated (eq 1), and the following cutoffs were used to classify ICC values: an ICC ≥ 0.75 indicates excellent stability, 0.51–0.74 indicates good stability, 0.40–0.50 indicates fair stability, and <0.40 indicates poor stability.^7,25^ Metabolomic bin regions were classed as stable if the ICC value was ≥0.51. Stable bin regions were carried forward for additional analysis and collectively are referred to as the stable urinary NMR metabolome.

### Longitudinal Structural Equation Modeling (SEM)

Longitudinal SEM was performed on stable bin regions using the R Package OpenMx (version 2.9.9).^26^ To establish regularity and randomness of sampling, means and variances were examined to ensure equality across twin order and zygosity groups (Supporting Information Table S-3). A saturated Cholesky decomposition model was performed to examine time-specific etiology, explaining whether new sources of variance emerge over time while also modeling variance through successive traits onto the same trait at each new time point.^27^ The saturated model assumes that genetic variation of each variable is determined by a genetic component underlying that variable, as well as all other variables ordered before it in the model. In SEM, standardized path coefficients are calculated by multiplying the path coefficient matrix by the inverse of the standard deviation. Standardization allows path coefficients to be compared, assessing the relative effects of the variables within the fitted regression model. Standardized path coefficients are squared to derive the proportion of variance.

### Conservation of the Metabolomic Profile

To measure conservation of an individual’s stable urinary NMR metabolomic profile, conservation indices were created.^15^ Several steps were carried out: (1) intra- and interindividual metabolomic profile correlations were calculated; Pearson’s correlations were performed between an individual’s baseline profile and all participants’ profiles at 2 months (*n* = 128); (2) correlations were ranked; an individual’s intraindividual correlation was ranked against the interindividual correlations of that individual with all other participants; (3) conservation indices were calculated using the formula 1-((rank(i)-1)/(N-1)).^15^ To compare our results to previously published methodologies,^15^ the conservation index of the entire metabolomic profile (400 bins) was recreated using intra- and interindividual metabolomic profile correlations that were weighted using longitudinal bin correlations. Longitudinal Pearson’s correlations were performed between the same metabolomic bin region at baseline and 2 months. Pearson’s correlations were controlled for age, sex, FFM, and HEI scores using the R package ppcor (version 1.1). Weighting was applied to metabolomic profile correlations using the R package pysch (version 2.0.7).

## Results

### Characteristics of the Cohort and the Stability of the Metabolomic Profile

This study included 88 MZ and 40 DZ twins, including 58 males and 70 females. The cohort had a mean age of 35 years (±13), a mean height of 170.5 cm (±8.5), a mean body mass of 70.4 kg (±11.4), and a mean BMI of 24.2 kg/m^2^ (±3.1) (Table 1). ICC values for 400 urinary bin regions ranged from 0.00 to 0.65, and the median ICC was 0.39 (±0.17). Pearson’s correlations ranged −0.22–0.59, and the median value was 0.16 (±0.17). Metabolomic bin regions classed as having good conservation (ICC ≥ 0.51) are presented in Table 2. Twenty percent of the urinary NMR metabolomic profile (81 bin regions) had good stability over 2 months and collectively make up the stable urinary NMR metabolome (Table 2). The contribution of sex, age, FFM, and HEI scores to variance in each stable metabolomic bin region is reported in Table 2. Age and diet quality (HEI score) significantly contributed to variance in 44% (36 bin regions) and 33% (27 bin regions) of the stable NMR metabolome, respectively. The median proportion of variance (*R*^2^) explained by age and HEI score was 10.9 and 4.8%, respectively. Sex and FFM significantly contributed to variance in fewer regions (10 and 7%) but the median proportion of variation explained by these covariates was 15 and 9.5%, respectively.

**Table 1:** Descriptives of the Cohort^a^

		total (*n* = 128)	MZ (*n* = 88)	DZ (*n* = 40)
gender	male	58	38	20
	female	70	50	20
age	years	35 (13)	37 (12)	32 (12)
height	cm	170.5 (8.5)	169.7 (8.0)	172.4 (9.3)
waist	cm	80.0 (8.7)	79.8 (9.6)	80.6 (6.3)
hip	cm	97.9 (7.4)	97.6 (7.7)	98.5 (6.6)
body fat	%	25.7 (9.5)	25.8 (9.6)	25.3 (9.5)
body mass	kg	70.4 (11.4)	69.5 (12.1)	72. 4 (9.6)
BMI	kg/m^2^	24.2 (3.1)	24.1 (3.3)	24.4 (2.5)

a All values are mean (±SD). *n*, number of observations; MZ, monozygotic; DZ, dizygotic; cm, centimeters; %, percentage; kg, kilograms; BMI, body mass index; and m^2^, meters squared.

**Table 2:** Proportion of Variance Explained by Significant Covariates and ICC for the Stable Urinary NMR Metabolome^a^

bin (ppm)	sex % (β)	*p*-value	age % (β)	*p*-value	FFM % (β)	*p*-value	HEI	*p*-value	ICC
3.35			7.1 (0.27)	0.021			12.1 (0.34)	<0.001	0.645
2.51	19.1 (0.87)	<0.001	8.1 (0.29)	0.019	10.1 (−0.31)	0.044			0.626
8.83									0.625
1.71									0.624
7.29							4.1 (0.20)	0.045	0.622
1.19									0.621
1.89									0.617
9.11									0.615
6.91									0.613
6.97							6.1 (0.24)	0.015	0.609
2.33			11.1 (0.34)	0.001			5.1 (−0.22)	0.028	0.604
1.25			8.1 (−0.29)	0.010					0.594
1.73									0.594
7.61			13.1 (0.37)	0.001			5.1 (0.22)	0.016	0.593
7.81			14.1 (0.37)	0.001			4.1 (0.20)	0.028	0.593
3.55					8.1 (-0.29)	0.045			0.591
3.41	8.1 (−0.58)	0.024							0.591
7.63			13.1 (0.37)	0.001			5.1 (0.23)	0.014	0.590
2.85									0.588
2.65	17.1 (0.83)	0.001	10.1 (0.32)	0.006	10.1 (−0.31)	0.043			0.587
7.27			16.1 (0.40)	<0.001					0.586
7.83			14.1 (0.38)	0.001			7.1 (0.25)	0.007	0.585
2.17									0.584
7.53			11.1 (0.33)	0.002					0.583
7.55			15.1 (0.39)	<0.001			7.1 (0.27)	0.004	0.578
1.47							3.1 (−0.17)	0.044	0.578
2.29									0.578
3.01									0.566
3.15	6.1 (−0.48)	0.039							0.566
3.03	20.1 (−0.90)	<0.001	20.1 (−0.45)	<0.001	19.1 (0.44)	<0.001			0.565
2.61									0.565
1.17							3.1 (0.17)	0.044	0.565
2.27			6.1 (0.25)	0.016					0.550
1.31			6.1 (−0.25)	0.019					0.563
2.43							4.1 (−0.20)	0.028	0.562
1.97									0.562
3.99			12.1 (−0.35)	<0.001			4.1 (−0.19)	0.048	0.560
7.35			16.1 (0.40)	<0.001					0.555
6.89									0.552
7.39			9.1 (0.30)	0.003					0.551
7.41			18.1 (0.42)	<0.001					0.548
2.67	18.1 (0.86)	<0.001			9.1 (−0.30)	0.038			0.547
0.97									0.546
1.93							7.1 (−0.27)	0.004	0.545
3.13									0.544
8.77									0.543
8.53			7.1 (0.27)	0.008					0.541
3.95			8.1 (0.29)	0.007			5.1 (0.22)	0.013	0.540
2.59							3.1 (0.18)	0.04	0.539
6.85									0.538
0.99			6.1 (−0.24)	0.016					0.537
0.95			11.1 (−0.34)	0.001			4.1 (−0.20)	0.038	0.537
3.93									0.536
3.19							6.1 (−0.24)	0.014	0.535
2.07							5.1 (−0.23)	0.013	0.534
2.53	13.1 (0.73)	0.003	6.1 (0.25)	0.025					0.533
2.41									0.532
2.05			5.1 (−0.21)	0.04			5.1 (−0.22)	0.014	0.530
2.03							5.1 (−0.21)	0.013	0.530
1.09									0.529
0.83			5.1 (-0.23)	0.024			4.1 (−0.20)	0.027	0.529
2.25			16.1 (0.40)	<0.001					0.528
2.35			6.1 (−0.25)	0.016			4.1 (−0.19)	0.044	0.526
6.83							6.1 (0.24)	0.011	0.526
2.31									0.525
0.89									0.524
1.27			5.1 (−0.22)	0.024					0.523
1.01									0.523
2.91			4.1 (−0.20)	0.049					0.522
7.33			17.1 (0.42)	<0.001					0.518
7.43			15.1 (0.38)	<0.001					0.517
7.37			13.1 (0.36)	<0.001					0.517
1.35					6.1 (0.25)	0.045			0.516
1.87							4.1 (−0.19)	0.039	0.516
7.65			13.1 (0.36)	<0.001			11.1 (0.32)	<0.001	0.515
2.69									0.515
3.45									0.514
3.05	7.1 (−0.55)	0.04	16.1 (−0.40)	<0.001					0.513
2.13							6.1 (−0.24)	0.014	0.510
1.77									0.510
3.83			7.1 (−0.26)	0.005					0.510

a %: *R*^2^ value calculated the proportion of variance explained by significant fixed effects. β: fixed effect parameter estimate; p-value: FDR (α < 0.05) corrected *p-*values from ANOVAs on the mixed models; ICC: intraclass correlations with significant covariates included as fixed effects; ppm: parts per million; FFM: fat-free mass; HEI: healthy eating index. *n* = 128 individuals.

### Contribution of Genetic and Environmental Influences to the Stable Urinary NMR Metabolome

Longitudinal variation in the stable urinary NMR metabolome was examined using a Cholesky decomposition model. The longitudinal Cholesky decomposition model permits examination of genetic or environmental influences that emerge at different times. Squared standardized path coefficients and confidence intervals for the top 10 most stable bin regions are reported in Table 3. Genetic (A1) and shared environmental factors (C1) present at baseline and persisting over time accounted for covariance in the metabolomic bin regions at each visit. A smaller proportion of genetic and shared environmental variances was unique to bin regions at each visit but did not influence variation at the next time point. For example, bin 3.35 ppm, the most stable metabolomic region, had a shared environmental factor common with all three visits (C1) that accounted for 0.40, 0.36, and 0.38 of variation at each time point, respectively. Three percent of the shared environmental variance (1% of total variance) at visit 3 was explained by a factor common with visits 2 and 3 only (C2), and none of the variance was explained by a factor unique to visit 3 (C3). For the top 10 stable urinary bin regions, 8 had a strong shared environmental factor common with all three visits, whereas 2 (bins 1.71 and 1.89 ppm) had a strong genetic factor common across visits. The Cholesky decomposition model estimates for the entire stable metabolome are reported in Supporting Information Table S-2.

**Table 3:** Cholesky Decomposition Squared Standardized Path Coefficients, Saturated Model^a^

bin (ppm)	A1	A2	A3	C1	C2	C3	E1	E2	E3
3.35	0.06 (0.00, 0.53)			0.40 (0.00, 0.62)			0.54 (0.36, 0.75)		
	0.08 (0.00, 0.57)	0.00 (0.00, 0.21)		0.36 (0.00, 0.62)	0.06 (0.00, 0.23)		0.08 (0.01, 0.22)	0.42 (0.28, 0.60)	
	0.00 (0.00, 0.38)	0.00 (0.00, 0.18)	0.00 (0.00, 0.17)	0.38 (0.00, 0.57)	0.01 (0.00, 0.15)	0.00 (0.00, 0.12)	0.15 (0.05, 0.33)	0.10 (0.03, 0.22)	0.36 (0.23, 0.52)
2.51	0.05 (0.00, 0.58)			0.47 (0.00, 0.66)			0.48 (0.31, 0.70)		
	0.00 (0.00, 0.55)	0.04 (0.00, 0.25)		0.42 (0.00, 0.62)	0.00 (0.00, 0.22)		0.05 (0.00, 0.17)	0.49 (0.32, 0.67)	
	0.01 (0.00, 0.51)	0.02 (0.00, 0.28)	0.00 (0.00, 0.20)	0.42 (0.00, 0.62)	0.00 (0.00, 0.19)	0.00 (0.00, 0.17)	0.06 (0.00, 0.18)	0.02 (0.00, 0.11)	0.47 (0.31, 0.65)
8.83	0.15 (0.00, 0.51)			0.32 (0.02, 0.56)			0.53 (0.35, 0.76)		
	0.00 (0.00, 0.31)	0.00 (0.00, 0.32)		0.48 (0.03, 0.65)	0.01 (0.00, 0.29)		0.04 (0.00, 0.15)	0.46 (0.31, 0.66)	
	0.01 (0.00, 0.30)	0.00 (0.00, 0.32)	0.00 (0.00, 0.23)	0.28 (0.00, 0.50)	0.02 (0.00, 0.26)	0.00 (0.00, 0.21)	0.22 (0.08, 0.43)	0.03 (0.00, 0.12)	0.44 (0.28, 0.63)
1.71	0.31 (0.00, 0.73)			0.29 (0.00, 0.64)			0.41 (0.26, 0.60)		
	0.35 (0.00, 0.67)	0.01 (0.00, 0.21)		0.16 (0.00, 0.58)	0.01 (0.00, 0.21)		0.00 (0.00, 0.06)	0.47 (0.32, 0.65)	
	0.43 (0.00, 0.75)	0.09 (0.00, 0.34)	0.00 (0.00, 0.25)	0.11 (0.00, 0.58)	0.00 (0.00, 0.28)	0.00 (0.00, 0.22)	0.00 (0.00, 0.06)	0.02 (0.00, 0.09)	0.36 (0.23, 0.54)
7.29	0.16 (0.00, 0.62)			0.33 (0.00, 0.64)			0.50 (0.33, 0.72)		
	0.10 (0.00, 0.65)	0.23 (0.00, 0.45)		0.18 (0.00, 0.53)	0.03 (0.00, 0.27)		0.10 (0.02, 0.24)	0.35 (0.22, 0.55)	
	0.00 (0.00, 0.42)	0.02 (0.00, 0.32)	0.00 (0.00, 0.23)	0.23 (0.00, 0.55)	0.11 (0.00, 0.37)	0.00 (0.00, 0.00)	0.11 (0.02, 0.28)	0.13 (0.03, 0.29)	0.40 (0.26, 0.57)
1.19	0.09 (0.00, 0.60)			0.37 (0.00, 0.62)			0.54 (0.37, 0.76)		
	0.45 (0.00, 0.70)	0.00 (0.00, 0.41)		0.11 (0.00, 0.64)	0.01 (0.00, 0.37)		0.03 (0.00, 0.11)	0.41 (0.27, 0.60)	
	0.13 (0.00, 0.53)	0.00 (0.00, 0.39)	0.00 (0.00, 0.28)	0.05 (0.00, 0.49)	0.16 (0.00, 0.40)	0.00 (0.00, 0.00)	0.07 (0.01, 0.22)	0.08 (0.01, 0.23)	0.51 (0.35, 0.71)
1.89	0.31 (0.00, 0.71)			0.26 (0.00, 0.62)			0.43 (0.28, 0.64)		
	0.36 (0.00, 0.63)	0.00 (0.00, 0.25)		0.06 (0.00, 0.50)	0.03 (0.00, 0.24)		0.03 (0.00, 0.14)	0.52 (0.35, 0.71)	
	0.44 (0.00, 0.64)	0.00 (0.00, 0.29)	0.00 (0.00, 0.00)	0.02 (0.00, 0.43)	0.00 (0.00, 0.23)	0.00 (0.00, 0.18)	0.04 (0.00, 0.16)	0.02 (0.00, 0.11)	0.48 (0.32, 0.67)
9.11	0.14 (0.00, 0.53)			0.32 (0.00, 0.56)			0.54 (0.36, 0.76)		
	0.01 (0.00, 0.31)	0.02 (0.00, 0.37)		0.40 (0.00, 0.60)	0.00 (0.00, 0.27)		0.07 (0.01, 0.20)	0.50 (0.33, 0.71)	
	0.07 (0.00, 0.36)	0.01 (0.00, 0.40)	0.00 (0.00, 0.28)	0.25 (0.00, 0.48)	0.00 (0.00, 0.24)	0.00 (0.00, 0.20)	0.24 (0.09, 0.45)	0.01 (0.00, 0.10)	0.41 (0.26, 0.62)
6.91	0.14 (0.00, 0.58)			0.31 (0.00, 0.60)			0.55 (0.37, 0.78)		
	0.01 (0.00, 0.50)	0.18 (0.00, 0.40)		0.17 (0.00, 0.50)	0.00 (0.00, 0.00)		0.11 (0.02, 0.28)	0.53 (0.35, 0.76)	
	0.00 (0.00, 0.38)	0.01 (0.00, 0.23)	0.00 (0.00, 0.00)	0.33 (0.00, 0.53)	0.00 (0.00, 0.24)	0.00 (0.00, 0.00)	0.07 (0.01, 0.21)	0.11 (0.02, 0.26)	0.48 (0.33, 0.66)
6.97	0.12 (0.00, 0.58)			0.31 (0.00, 0.58)			0.57 (0.38, 0.80)		
	0.00 (0.00, 0.47)	0.00 (0.00, 0.31)		0.31 (0.00, 0.51)	0.00 (0.00, 0.28)		0.11 (0.02, 0.28)	0.57 (0.38, 0.76)	
	0.01 (0.00, 0.52)	0.00 (0.00, 0.31)	0.00 (0.00, 0.02)	0.39 (0.00, 0.58)	0.00 (0.00, 0.00)	0.00 (0.00, 0.16)	0.10 (0.02, 0.25)	0.06 (0.01, 0.17)	0.45 (0.31, 0.61)

a Squared standardized path coefficients and 95% confidence intervals are presented. A1–A3, additive genetic factors; C1–C3, shared environmental factors; and E1–E3, unique environmental factors. All models were controlled for age and sex. *n* = 128 individuals.

### NMR Urinary Metabolomic Profile Conservation

Conservation indices were calculated for the stable urinary NMR metabolome (Table 4). Thirty-four percent of individuals had an excellent conservation index of 1.00, meaning they were most similar to themselves after 2 months; 91% of individuals had a conservation index ≥0.70, meaning these individuals ranked among the 30% highest correlations with all other profiles; and only 9% of individuals had a lower conservation index (<0.70). Using a weighted method on the entire metabolomic profile, 90% of individuals had a conservation index ≥0.70 and 10% of individuals had a lower conservation index. Conservation indices calculated using the stable urinary NMR metabolome only and the weighted method yielded similar results; however, the stable method had more individuals with excellent indices.

**Table 4:** NMR Urinary Metabolomic Conservation Indices

conservation index	stable metabolome (81 bins)	weighted metabolomic profile (400 bins)
	*n* (%)	*n* (%)
1.00	44 (34)	40 (31)
0.90–0.99	51 (40)	55 (43)
0.70–0.89	22 (17)	20 (16)
<0.70	11 (9)	13 (10)

## Discussion

Twenty percent of the urinary NMR metabolomic profile is stable over a 2 month period. Genetic and shared environmental influences present at baseline persisted and consistently accounted for sources of variation across time. The stable urinary NMR metabolome had a high conservation index for 91% of the cohort. Sex, age, FFM, and diet quality were associated with many regions of the stable urinary NMR metabolome but the contribution of covariates to total variance was relatively low ranging from 0 to 20.3%. The stable urinary NMR metabolome, composed of 81 bin regions, provides an effective method to distinguish individuals from one another and to measure or monitor metabolomic conservation over time.

This research aims to understand what factors contribute toward stability in the urine NMR metabolomic profile. Having identified a stable component of the urinary NMR metabolomic profile, this study demonstrates that both genetic and shared environmental factors contribute to stability over time. Heritability estimates ranged from 0.00 to 0.69 across the three study visits with a median heritability of 0.16. A genetic factor common with visit 1 explained on average 82% of heritability at visit 2 and 79% at visit 3. This agrees with previous research suggesting the presence of a genetic component influencing an individual’s “metabolomic fingerprint” over time.^12^ Yousri et al.^15^ showed that heritability and stability of metabolites over 7 years were highly correlated, suggesting that metabolites are more conserved because of genetic influences. The authors suggested that metabolites with high stability and low heritability were conserved as a result of environmental factors which could be diet or lifestyle related.^15^ We previously identified a collection of reproducible urinary metabolomic regions that were consistently correlated to habitual diet quality over time in both MZ and DZ twins.^20^ The same diet-associated metabolomic regions were captured in the stable NMR metabolome in this study, suggesting the influence of habitual diet on conservation. In this study, shared environmental estimates ranged from 0.00 to 0.56 with a median estimate of 0.18. A shared environmental factor common with visit 1 explained on average 81% of shared environmental influences at visit 2 and 85% at visit 3. In adult twins living apart, the shared environment may represent lasting influences of their time cohabiting or similar current living environments, including the same/similar neighborhood, exposure to similar pollutants, or shared diet and lifestyle. Significantly correlated metabolites between spouses who share a household indicate that shared environment contributes to similarities in the metabolome.^28^ This longitudinal modeling demonstrates that familial factors, composed of genetics and shared environment, influence variation in the urinary NMR metabolome over time.

To the best of our knowledge, two studies to date have examined variation in metabolomic profiles over time using twins.^16,29^ In a cohort of 56 MZ and 21 DZ twin pairs, familial factors contributed ∼30% of variation in urinary metabolites.^16^ Thirty-four MZ twins donated samples twice over 4 months, which allowed decomposition of the remaining nonfamilial variation (i.e., unique environment) into individual environment, individual-visit, and common-visit components. The authors describe individual environment as a stable component that captures long-term lifestyle factors such as diet, culture, and social factors that are unique to an individual.^16^ The visit components capture variation between sample collection time points. In this study, we have three urine samples per person (MZ and DZ twins) collected over time, which when combined with a multivariate modeling approach permits decomposition of covariation between time points into genetic, shared, and unique environmental factors. Although some metabolomic regions identified as highly stable are similar across both studies (e.g., bin 3.35 ppm), the variance estimates are not comparable. Another study reported heritability estimates for 901 serum metabolites at three time points collected over an 18 year period.^29^ However, Long et al.^29^ reported univariate heritability estimates for individual time points and categorized metabolites as consistently heritable if the coefficient of variation between time points was <0.50. The shared environment also contributed to variation in consistently heritable serum metabolites but shared environment consistency was not reported.^29^ Similarly, this study reports heritability at three visits but longitudinal Cholesky decomposition modeling allows time-specific sources of variation to emerge and allows potential short-term changes in environment to be identified. This study extends the existing knowledge and demonstrates that genetic and shared environmental factors exert a stable and pervasive influence on urinary metabolites, which contributes to the conservation and uniqueness of an individual’s stable urinary NMR metabolome.

We examined several covariates to elucidate the factors contributing to metabolomic stability. Many factors including age, sex, and weight are commonly adjusted covariates in studies but these may also contribute toward stability. In this study, some metabolomic regions were more strongly influenced by covariates than others. For example, 32% of variation in region 3.03 ppm was explained by age, sex, and FFM. Region 3.03 ppm represents a clear peak for the metabolite creatinine, an end product of creatine metabolism produced at a steady rate in the body.^30^ Blood creatine^15^ and urinary creatinine^4^ are stable, and creatinine production decreases with age, varies with sex, and a positive relationship exists with FFM.^31,32^ In this study, covariates accounted for a larger proportion of variance in stable urinary bin regions than previously reported.^4^ Age was associated with the largest number of stable regions and was also the covariate with the highest contribution to variance in a stable bin. Biologically significant changes occur with aging, and the majority of age-associated metabolites are related to lipid and amino acid pathways.^33^ Overall, covariates or their combinations explained some of the variance in the stable metabolome but future research should examine other covariates contributing to variance, such as gut microbiota and physical activity.

This research also demonstrates that the stable urinary NMR metabolome is distinguishable and conserved over time. The stable NMR metabolome showed good conservation for 91% of the cohort (index ≥ 0.70). Plasma metabolome conservation was reported in the KORA and TwinsUK cohorts,^15^ where 95% of individuals had conversation indices > 0.83 and > 0.78, respectively. Differences between cohorts are likely due to the different biofluids; that is, urinary metabolites are more variable and sensitive to day-to-day changes and dietary intake. Thus, urinary profiles may provide additional, valuable information about long-term dietary influences on metabolomic conservation. Yousri et al.^15^ weighted metabolome conservation indices using longitudinal metabolite intracorrelations and demonstrated improved metabolomic conservation in their cohort.^15^ In this study, the stable method and weighted method yielded similar results; however, the stable method had more individuals with excellent indices. Similarity in results demonstrates that using ICCs to identify regions with low intraindividual variation is an effective method to distinguish individuals and supports evidence that not all regions are equally informative for identification of individuals as themselves at a later time. Across all studies, some individual’s profiles were less well conserved and may signify a significant lifestyle or health status change, such as antibiotic treatment, or pregnancy/breastfeeding.^14^ Lacruz et al.^17^ demonstrated that poor metabolome conservation is associated with an increase in all-cause mortality risk independent of several other health parameters.^17^ Evidence of change in an individual’s stable metabolome could support early intervention for an illness or disease. However, we must further understand intraindividual metabolomic variation and begin to identify the factors contributing to metabolite stability before we can use profiles to inform diet and lifestyle change or alter disease trajectory.

Strengths and limitations of this study should be considered when interpreting the results. This classic twin study cohort permits analysis of the genetic and environmental factors influencing variance in traits. Our cohort is healthy, and the sample size is small; to overcome some limitations, we ensured that twin assumptions were not violated and incorporated covariates in all models. Sample numbers must still be considered when interpreting model estimates. Study design and sample size permitted controlled sample collection at multiple time points, reducing the impact of preanalytic sample collection factors. The metabolomic technique (NMR) used in this study has high analytical reproducibility and low interlaboratory variation. However, NMR represents only a portion of the metabolome and spectral binning was performed with limitations including reduced resolution and peaks shifting between bins. We acknowledge that this research is exploratory and validating results in larger cohorts would strengthen our findings.

To conclude, this study shows that 20% of the urinary NMR metabolomic profile is stable over 2 months. The stable urinary NMR metabolome is influenced by a combination of genetic and shared environmental factors, which exert a stable and pervasive influence on metabolomic regions over time. Factors including age, sex, FFM, and diet quality are associated with stable metabolomic regions. The stable urinary NMR metabolome of an individual is recognizable and conserved over time. If we know an individual’s conserved metabolome, then deviations from a stable state may be indicative of disease and potentially provide novel information on biomarkers of diseases. Further work should try to identify the remaining influences (e.g., gut microbiota, stress, etc.) contributing toward conservation of the stable metabolome. This knowledge may inform personalized recommendations that optimize health and prevent disease based on an individual’s stable metabolome.

## Supporting Information Available

UCD twin study workflow design (Figure S-1) (PDF)
Power to detect significant parameters in the UCD twin study cohort (Table S-1); and decomposition squared standardized path coefficients for the stable urinary NMR metabolome, saturated model (Table S-2); difference in log-likelihood Δχ^2^ for tests concerning means and variances of the stable urinary NMR metabolome (Table S-3) (XLSX)

## Abbreviations Used

A: additive genetic effects
BMI: body mass index
C: shared environmental effects
DZ: dizygotic
E: unique environmental effects
FDR: false discovery rate
FFM: fat-free mass
GC: gas chromatography
HEI: healthy eating index
ICC: intraclass correlation
LC: liquid chromatography
MS: mass spectrometry
MZ: monozygotic
NMR: nuclear magnetic resonance
NOESEY: nuclear Overhauser spectroscopy
ppm: parts per million
SEM: structural equation modeling
