# Supplementary material for: Genetic and Environmental Contributions to Variation in the Stable Urinary NMR Metabolome over Time: A Classic Twin Study
Source: J Proteome Res. 2021 Jul 26;20(8):3992–4000. doi: 10.1021/acs.jproteome.1c00319 (PMC8397426; doi:10.1021/acs.jproteome.1c00319)
Supplement: Supplementary file 1 — pr1c00319_si_001.pdf [file pr1c00319_si_001.pdf]

Supporting Information

Title: Genetic and Environmental Contributions to Variation in the Stable Urinary NMR Metabolome Over Time: A Classic Twin Study.

Authorship: *Kate M. Bermingham* [a], *Lorraine Brennan* [a], *Ricardo Segurado* [b], *Rebecca E. Barron* [a], *Eileen R. Gibney* [a], *Miriam F. Ryan* [a], *Michael J. Gibney* [a], *Aifric M. O'Sullivan* [a*].

***Corresponding author:** Aifric.OSullivan@ucd.ie

Table of contents

Table S-1. Power to detect significant parameters in the UCD twin study cohort.

Table S-2. Cholesky Decomposition squared standardized path coefficients for the stable urinary NMR metabolome - Saturated Model

Table S-3. Difference in log likelihood $\Delta\chi2$ for tests concerning means and variances of the stable urinary NMR metabolome

Figure S-1. UCD twin study workflow design

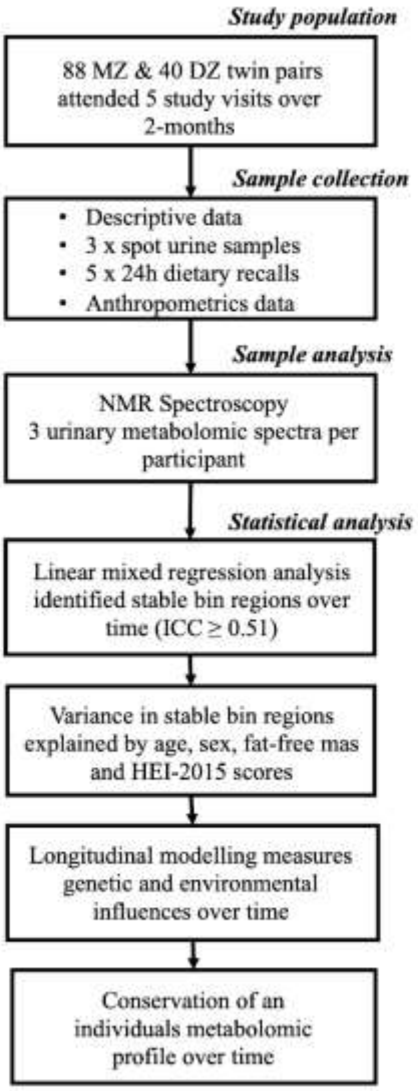

Figure S-1. UCD twin study workflow design
